# The association between pubertal status and depressive symptoms and diagnoses in adolescent females: A population-based cohort study

**DOI:** 10.1371/journal.pone.0198804

**Published:** 2018-06-18

**Authors:** Gemma Lewis, Konstantinos Ioannidis, Anne-Laura van Harmelen, Sharon Neufeld, Jan Stochl, Glyn Lewis, Peter B. Jones, Ian Goodyer

**Affiliations:** 1 Division of Psychiatry, University College London, London, United Kingdom; 2 Department of Psychiatry, University of Cambridge, Cambridge, United Kingdom; CUNY, UNITED STATES

## Abstract

**Background:**

There is an association between puberty and depression, but many things remain poorly understood. When assessing puberty in females, most studies combine indicators of breast and pubic hair development which are controlled by different hormonal pathways. The contributions of pubertal timing (age at onset) and pubertal status (stage of development, irrespective of timing) are also poorly understood. We tested the hypothesis that stage of breast development in female adolescents, controlled largely by increased estradiol, would be more strongly associated with depression than pubic hair development which occurs in both males and females, and is controlled by adrenal androgens. We investigated whether this association was independent of pubertal timing.

**Methods:**

ROOTS is an ongoing cohort of 1,238 adolescents (54% female) recruited in Cambridgeshire (UK) at age 14.5, and followed-up at ages 16 and 17.5. Depression was assessed using the Mood and Feelings Questionnaire (MFQ) and clinical interview. Breast and pubic hair development were assessed at 14.5, using Tanner rating scales.

**Results:**

For each increase in Tanner breast stage at 14.5, depressive symptoms increased by 1.4 MFQ points (95% CI 0.6 to 2.3), irrespective of age at onset. Pubic hair status was only associated with depressive symptoms before adjustment for breast status, and was not associated with depression in males. The same pattern was observed longitudinally, and for depression diagnoses.

**Limitations:**

We did not directly measure hormone levels, our findings are observational, and the study had a relatively low response rate.

**Conclusions:**

Females at more advanced stages of breast development are at increased risk of depression, even if their age at pubertal onset is not early. Alongside social and psychological factors, hormones controlling breast but not pubic hair development may contribute to increased incidence of female depression during puberty.

## Introduction

Adult depression affects twice as many women as men [1]. In childhood, the prevalence of depression is similar in boys and girls but around the age of 12–13, incidence increases sharply in females [2]. There is evidence that this increase is more strongly associated with pubertal development than chronological age [3]. However, although studies report an association between pubertal development and depression, many things remain poorly understood.

First, the influences of pubertal timing (age at onset) and pubertal status (stage of development, irrespective of timing) are poorly understood. The pubertal timing hypothesis states that early onset puberty is a risk factor for depression. The pubertal status hypothesis is that, when puberty occurs, it increases risk of depression irrespective of whether timing is early or late. An improved understanding of the relative influences of pubertal timing and status could advance our knowledge of depression aetiology, and the gender difference in prevalence. Pubertal timing is being considered a target for preventive interventions, amid concerns that age of onset is falling in many countries [4,5]. However, later developing girls might still be at increased risk of depression when they reach more advanced pubertal stages (irrespective of timing).

Findings on the association between pubertal timing and depression are inconsistent, and a recent review rated the quality of evidence as low [6]. One study has used Mendelian Randomisation, which should eliminate confounding, and found that earlier age at menarche was associated with depressive symptoms at age 14, but not at ages 17 or 19 [7]. At age 14, those who experience early puberty will also be at a more advanced pubertal stage. This will be the case until all other participants have caught up. More advanced pubertal stage might therefore be on the causal pathway from pubertal timing to depressive symptoms. This might explain why an association was observed at age 14 but not at ages 17 or 19 [7]. To our knowledge, only three studies have compared pubertal timing and status [8–10]. Two found that pubertal status accounted for effects of pubertal timing [9,10], and one found that both were independently associated with depression [8].

It is also unclear how an early onset of puberty would contribute to the gender difference in depression, which persists throughout adult life. The persistence of the gender difference is more consistent with the hypothesis that, when females experience puberty, changes occur that increase their risk of depression throughout the lifecourse. One hypothesis is that increased levels of certain gonadal hormones which, from puberty onwards, are much higher in females, affect areas of the brain that process emotional information, possibly via effects on serotonin and the HPA axis [11]. Estrogens increase markedly in females (but not males) during puberty, and remain higher during the reproductive years, declining postmenopause [12]. There is evidence that the prevalence of depressive symptoms follows a similar pattern [3,13]. The potential role of female gonadal hormones was supported in a recent study which found an association between depression and hormonal contraceptives, particularly in adolescents [14].

Gonadal hormones are difficult to investigate directly, due to their monthly and circadian cycles. A recent review found some evidence for a positive association between estradiol and depressive symptoms, but most studies were small (<339) and cross-sectional [15]. In larger studies that do not have direct assessments of hormones, physical measures of pubertal development can be used as a proxy [16]. However, potential inferences about underlying hormones have been limited in these studies by the fact that most use composite pubertal ratings that combine stage of breast development with stage of pubic hair development. Breast development is controlled by increased levels of gonadal hormones, particularly estradiol (gonadarche) whereas pubic hair development is controlled by dehydroepiandrosterone (DHEA), an adrenal androgen (adrenarche). One study of pubertal timing and depression compared breast and pubic hair development in a Chinese cohort, and found that timing of breast, but not pubic hair development, was associated with increased depressive symptoms [17]. This supports the hypothesis that breast development, and possibly gonadal hormones, are more important in the development of depressive symptoms than pubic hair development and adrenal androgens. However, no study of pubertal status has compared associations between depression and breast versus pubic hair development.

In this study we used a population-based cohort to compare associations between breast versus pubic hair status and depression in female adolescents. We also compared pubertal status and pubertal timing. To test the hypothesis that stage of breast development is a marker for the emergence of the gender difference in depression that is unique to females, we also examined the association between pubic hair status and depression in males.

## Method

### Participants

The ROOTS study is an ongoing investigation of adolescents recruited from 18 secondary schools in Cambridgeshire (UK) [18]. 3762 adolescents were invited to participate, and 1238 (33%) agreed (54% female). Data were collected at three time-points, when adolescents were 14.5, 16.0, and 17.5 years of age. This study uses data from Times one and three which had information on both depressive symptoms and diagnoses. At Time 3, 1074 adolescents provided data (87% retention). All procedures contributing to this work comply with the ethical standards of the relevant national and institutional committees on human experimentation and with the Helsinki Declaration of 1975, as revised in 2008. All participants and parents / guardians provided written informed consent. Ethical approval for the ROOTS study was granted by Cambridgeshire 2 REC, in accordance with the Declaration of Helsinki and Good Clinical Practice guidelines.

### Measures

*Depression*. At each time-point, adolescents completed the long (33-item) Mood and Feelings Questionnaire (MFQ) [19]. The MFQ assesses DSM based depressive symptoms over the past 2 weeks on a three-point Likert scale ranging from 0 to 2. Possible scores range from 0 to 66, with higher scores indicating more severe depressive symptoms. Internal consistency was high across time-points (alpha 0.89 to 0.93). Semi-structured diagnostic interviews were conducted by trained interviewers at Time one and three, using the Schedule for Affective Disorders and Schizophrenia for School-Age Children present and lifetime version (K-SADS-PL) [20]. The K-SADS-PL derives DSM-IV diagnoses of current and lifetime depression. Agreement of diagnoses between raters was high at both time-points (>95%).

*Pubertal status*. Adolescents rated their pubertal stage using schematic drawings accompanied by short descriptions, based on Tanner stages [21]. Reliability between Tanner stages and clinician ratings of pubertal stage has been found to be high [22]. The female questionnaire assessed stages of breast and pubic hair development; the male questionnaire stages of genital and pubic hair development. Each scale comprised five ordinal response categories: no development (1) to mature development (5). Adolescents were asked to tick the picture and description that best described them.

*DHEA*. Hormonal data were collected in the ROOTS study on DHEA but not estradiol. Few cohort studies have data on estradiol which is difficult to measure due to daily and monthly fluctuations. We used the DHEA data to explore the validity of the Tanner scales (see statistical analyses section). At age 14.5, adolescents provided morning and evening saliva samples for three consecutive days. They were asked to provide their morning sample as close as possible to 30 minutes after waking (after drinking only water). Their evening sample was provided at 8pm (after drinking only water for 30 minutes before). DHEA assay was measured by ELISA on 20-μl samples of saliva without extraction (antibody, Cambio). Intra-assay variation was 5.1%, inter-assay variation 7.4%. Results are reported in ng/ml.

*Possible confounders*. Pubertal timing was assessed in females only, using self-reported age at onset of menarche. We created a categorical variable (early = under 11 years; normal = 12 to 13 years; late = 14 years or over). Next we adjusted for childhood adversity (from birth to age 6) and indicators of socioeconomic position (social class and maternal education) as preceding variables that might have caused both pubertal status and depression [23]. Childhood adversity between birth and age 6 was assessed retrospectively by interviewing parents when adolescents were 14.5 years old, using the Cambridge Early Experiences Interview (CAMEEI) [24,25]. We used a latent class variable derived in a previous study [24]. Socioeconomic position was assessed using Acorn, a 5-category proxy of social and economic class derived from postcode data by CACI (http://www.caci.co.uk/products/product/acorn), and maternal education (highest qualification obtained, ranging from none to a higher degree). Data on BMI were collected from 930 adolescents who were weighed and measured at their school approximately six months after the baseline assessment.

### Data

The raw data and analysis code used for this manuscript is available from www.figshare.com using the DOI 10.6084/m9.figshare.6450191.

### Statistical analyses

All analyses were conducted in STATA 13, separately for females and males. Robust sandwich estimators which adjust standard errors to take account of skewness were used for continuous outcomes that were not normally distributed. Residual distributions were checked for normality.

#### DHEA: Associations with pubertal status and depressive symptoms

We tested whether DHEA was more strongly associated with pubic hair than breast or genital development, which would be expected if the Tanner scales were valid. First we used a series of univariable linear models to test whether each indicator of pubertal status (breast, genital and pubic hair status: continuous exposures) was associated with DHEA (continuous outcome). We then ran bivariable models including both indicators of pubertal status, to test whether associations between breast (girls) / genital (boys) status and DHEA were accounted for by pubic hair status. Next we tested associations between DHEA (continuous exposure) and depressive symptoms using linear regression.

#### Cross-sectional associations between pubertal status and depressive symptoms

For girls, two separate univariable models were initially tested, both with depressive symptoms as the outcome and 1) breast and 2) pubic hair status as exposures. Bivariable models including both breast and pubic hair status were then examined. The same models were run for boys (using genital and pubic hair status). Models were then adjusted for potential confounders. We did not examine cross-sectional associations with depression diagnoses because of lack of power (only 21 girls and 10 boys received a diagnosis of current depression at age 14.5).

#### Longitudinal associations between pubertal status and depressive symptoms and diagnoses

The same models were run longitudinally with depressive symptoms at age 17.5 the outcome. Next we tested longitudinal models with diagnoses of depression by age 17.5 as a binary outcome, using logistic regression. Girls with lifetime diagnoses of depression by age 14.5 were removed from these analyses to test incident diagnoses of depression by age 17.5. Associations with depression diagnoses were not examined in boys because of lack of power.

*Missing data*. We used multiple imputation by chained equations (MICE) to account for missing data because complete case analyses can introduce bias when data are not missing completely at random. We assumed missingness was dependent on observed data (missing at random) and imputed 25 datasets [26]. To predict missing data we used all variables in analysis models and all potential confounders in Table 1. We also included depressive symptoms at Time two, to enhance prediction of missing depressive symptom and diagnostic data at Time three. We then ran analyses across imputed datasets. Our primary analyses were conducted on an imputed dataset based on those with complete data on the exposure variables. This resulted in an imputed sample of 658 girls and 511 boys (i.e. we replaced missing data for all individuals who reported their stage of pubertal development). Imputing outcome and confounder data reduces the possibility of attrition bias and increases power and precision [27]. Complete case analyses make the unrealistic assumption that data are all ‘missing completely at random’ (MCAR). In the ROOTS study, missing data are associated with observed variables, increasing the plausibility of the ‘missing at random’ (MAR) assumption required for MICE. As we imputed over 40% of the sample, we present data on the complete case sample as a sensitivity analysis in the supplement (S3 Table and S4 Table).

**Table 1:** Characteristics of the study sample at baseline, according to breast (girls) and pubic hair (boys) status, all available data.

Characteristic	Breast status, girls			Pubic hair status, boys		
	I and II	III	IV and V	I and II	III	IV and V
Overall numbers in sample (%)	61 (9)	217 (33)	380 (57)	68 (10)	295 (45)	296 (45)
Early age at onset of menarche <12% (N)	2 (6)	20 (11)	64 (19)	n/a	n/a	n/a
Experienced childhood adversity % (N)	22 (37)	70 (34)	163 (46)	12 (35)	34 (32)	159 (43)
Low socioeconomic status % (N)	6 (10)	12 (26)	14 (53)	5 (14)	19 (17)	65 (17)
Low maternal education % (N)	17 (32)	45 (23)	78 (24)	9 (30)	20 (19)	80 (23)

### Role of the funding source

The funding source had no role in study design, data collection, data analysis, interpretation or writing of the report. The corresponding author had full access to all data used in the study, and had final responsibility for the decision to submit for publication.

## Results

### Descriptive statistics

Data on pubertal status were provided by 658 girls at Time one. Of these, 577 provided depression data at Time 3. Complete data on all variables were available for 367 girls (56%). Girls with missing data (n = 291) had higher MFQ scores at Time one and three. There were no differences according to exposure variables. However, girls with missing data were more likely to have mothers with lower education, and be of moderate/hard pressed social means.

Data on pubertal status were provided by 511 boys at Time one. Of these, 405 provided depression data at Time 3. Complete data on all variables was available for 288 boys (56% of total sample). Boys with missing data (N = 223) had higher MFQ scores at Time one and three. There were no differences on the exposure or other variables.

Table 1 shows characteristics of adolescents according to breast status for girls and pubic hair status for boys (characteristics according to pubic hair status for girls and genital status for boys were similar, available on request).

Subsequent analyses were conducted on imputed data to reduce risk of bias and increase power. The imputed sample included 658 girls (98% of the entire sample) and 511 boys (91% of the entire sample). Results from the complete case analyses showed the same pattern of associations and are presented in S3 Table and S4 Table.

#### DHEA: Associations with pubertal status and depressive symptoms

There was evidence of univariable associations between more advanced pubertal status and increased DHEA for girls and boys, S1 Table. In a multivariable model including both breast and pubic hair status, the association with breast status reduced (0.07, 95% CI -.00 to 0.09, p = .110), but for pubic hair status it remained (0.12, 95% CI 0.09 to 0.15, p = .005). The same pattern was observed for boys, S1 Table.

There was no evidence of an association between DHEA and depressive symptoms in girls or boys, S2 Table.

#### Cross-sectional associations between pubertal status and depressive symptoms

Change in depressive symptoms for a 1-stage increase in pubertal status is shown in Table 2. In univariable models, there was evidence for an association between more advanced breast status and increased depressive symptoms. There was weaker evidence of a similar association between pubic hair status and depressive symptoms. In a bivariable model containing both breast and pubic hair status the association with breast status remained, but the association with pubic hair status attenuated substantially. The association between breast status and depressive symptoms remained after adjusting for confounders. Pubertal timing was not associated with depressive symptoms after adjustment for pubertal status. There was no evidence of an association between pubic hair status and depressive symptoms in boys after adjustments for confounders.

**Table 2:** Cross-sectional associations between pubertal status (continuous exposure coded 1 to 5) and depressive symptoms (continuous outcome) at age 14.5 in girls (N = 658) and boys (N = 511).

Model	Change^a^ in depressive symptoms for a 1-stage increase in pubertal status (95% CI) p value		
	Girls		Boys
	Breast status	Pubic hair status	Pubic hair status
Model 1: Unadjusted	2·00 (1·09 to 2·92) <·0001	·99 (-·07 to 2·05) ·068	·42 (-·42 to 1·27) ·326
Model 2: Model 1 adjusted for other puberty measure^b^	1·90 (·93 to 2·88) <·0001	·42 (-·69 to 1·52) ·460	·56 (-·45 to 1·57) ·277
Model 3: Model 2 adjusted for age and pubertal timing^c^	1·74 (·75 to 2·73) <·0001	·40 (-·70 to 1·50) ·476	·58 (-·44 to 1·60) ·263
Model 4: Model 3 adjusted for possible confounders^d^	1·54 (.55 to 2·53) .002	·15 (-·93 to 1.23) .783	·46 (-.58 to 1.49) .387

^a^Change is represented by the unstandardized regression coefficient.

^b^In models with breast status as the initial exposure variable, adjustment was made for pubic hair status. In models with pubic hair status as the initial exposure variable, adjustment was made for breast status in girls and genital status in boys.

^c^Pubertal timing was included in models for girls only because this measure was unavailable for boys.

^d^Other possible confounders were childhood adversity, social class, maternal education, and BMI.

#### Longitudinal associations between pubertal status and depressive symptoms and diagnoses

Change in depressive symptoms for a 1-stage increase in pubertal status is shown in Table 3. In a bivariable model including breast and pubic hair status, there was evidence for a positive association between advanced breast but not pubic hair status at age 14.5 and higher depressive symptoms at age 17.5. The association between breast status and depressive symptoms remained after all adjustments. There was no evidence of an association between pubic hair status and depressive symptoms in boys after adjustments for confounders. The same pattern of associations was observed when depressive diagnoses by age 17.5 were the outcome variable, Table 3.

**Table 3:** Longitudinal associations between pubertal status (continuous exposure variable coded 1 to 5) and depressive symptoms / diagnoses (outcome variable) in girls (N = 658) and boys (N = 511). Longitudinal associations with depression diagnoses are presented for girls only (N = 586, those with lifetime depression diagnoses by the age of 14.5 excluded, N = 72).

**Linear regressions for depressive symptoms**	**Change**^a^ **in depressive symptoms for a 1-stage increase in pubertal status (95% CI) p value**		
	**Girls**		**Boys**
	Breast status	Pubic hair status	Pubic hair status
Model 1: Unadjusted	1·93 (·99 to 2·88) <·0001	·79 (-·22 to 1·81) ·124	1·17 (·16 to 2·17) ·023
Model 2: Model 1 adjusted for other puberty measure^b^	1·88 (·90 to 2·86) <·0001	·24 (-·79 to 1·26) ·654	1·11 (-·09 to 2·32) ·071
Model 3: Model 2 adjusted for age and pubertal timing^c^	2·02 (·99 to 3·05) <·0001	·26 (-·77 to 1·29) ·619	1·09 (-·11 to 2·28) ·075
Model 4: Model 3 adjusted for possible confounders^d^	1·64 (.60 to 2.69) .002	·03 (-·99 to 1.05) .961	.94 (-.26 to 2.14) .125
**Logistic regressions for depressive diagnoses**	**Odds ratio (95% CI) p value**		
	**Breast status**	**Pubic hair status**	**Pubic hair status**
Model 1: Unadjusted	1·50 (1·15 to 1·97) ·003	1·25 (0·92 to 1·70) ·152	n/a
Model 2: Model 1 adjusted for other puberty measure^b^	1·47 (1·11 to 1·94) ·006	1·12 (0·82 to 1·53) ·487	n/a
Model 3: Model 2 adjusted for age and pubertal timing^c^	1·47 (1·10 to 1·95) ·008	1·12 (0·82 to 1·53) ·486	n/a
Model 4: Model 3 adjusted for possible confounders^d^	1·41 (1·04 to 1·90) ·025	1·08 (·79 to 1·49) ·632	n/a

^a^Change is represented by the unstandardized regression coefficient.

^b^In models with breast status as the initial exposure variable, adjustment was made for pubic hair status. In models with pubic hair status as the initial exposure variable, adjustment was made for breast status in girls and genital status in boys.

^c^Pubertal timing was included in models for girls only because this measure was unavailable for boys.

^d^Other possible confounders were childhood adversity, social class, and maternal education.

## Discussion

We found evidence that girls at more advanced stages of breast development at age 14 had more depressive symptoms than girls at lower stages of breast development, irrespective of age at pubertal onset (pubertal timing). We also found that girls at more advanced stages of breast development at age 14 continued to report more depressive symptoms at 17.5 years of age. They were also more likely to meet criteria for incident clinical depression by age 17.5. We found no evidence that pubic hair development was associated with depressive symptoms and diagnoses, except before adjustment for breast development. This was supported by lack of evidence for an association between DHEA and depression (pubic hair development is controlled largely by DHEA). So, girls at more advanced stages of breast development were at increased risk of depression irrespective of their age at onset and level of pubic hair development / DHEA. There was no evidence that pubic hair development was associated with depressive symptoms in boys.

### Strengths and limitations

Strengths of this study include a prospective population-based sample, with self-report and some hormonal (DHEA) indicators of pubertal status. This allowed us to validate, to some extent, pubic hair status as a better indicator of adrenal hormones (DHEA) than breast status. We also examined depressive symptoms and clinical diagnoses.

This study has several limitations. We did not directly measure pubertal hormones other than DHEA and, in particular, we did not have access to any data on estradiol. Direct measures of gonadal hormones are difficult to get in large population based samples due to their circadian and monthly cycles. We used breast and pubic hair development as proxy variables, but there is some evidence that Tanner breast stage correlates only weakly with underlying hormones such as estrogen [28] although several studies report higher correlations [29]. However, the pattern of associations we observed for DHEA was consistent with our expectation that DHEA would be more strongly associated with breast than pubic hair development. Self-reported breast and pubic hair status may also be less reliable than clinician ratings. However, previous studies report strong to moderate associations between self-reported pubertal status and clinician assessments [22]. Future studies are required to establish the strength of the association between Tanner breast stage and gonadal hormones.

Another potential limitation of our study was that it was conducted quite late in the pubertal process. However, consistent with another UK cohort only a small proportion of female adolescents were in the most advanced stage of breast development, suggesting adequate power for our comparisons [30]. Although the average age of menarche is around 13 years of age [31], this would not have affected our ability to adjust for possible effects of pubertal timing because, according to the pubertal timing theory, those who started their periods earlier would still be at increased risk of depression later in adolescence and even in early adulthood [32]. The ROOTS study also had a relatively low response rate (33%) so if non-responders differed to responders on the exposure or outcome, this might have introduced selection bias. It would also reduce the generalizability of the sample to the population from which it was recruited.

Although we adjusted for several potential confounders, as with all observational studies, residual confounding is still a possibility. For example, we adjusted for Body Mass Index but unmeasured confounding by childhood adiposity might still have occurred. Randomised Controlled Trials (RCTs) and Mendelian Randomisation studies are superior designs for causal inferences. Depressive symptoms increase during the menopausal transition [33], however, RCTs of hormone therapy with estrogen and progesterone for menopausal women have produced inconsistent findings [34,35]. Findings from studies of older women might also not be generalizable to adolescents. Mendelian Randomisation studies are also inconsistent, with evidence of an association between age at menarche (as a proxy for estrogen exposure) and depressive symptoms in adolescents but not in older women [7,36]. More research on the association between estrogens and depression is therefore required, particularly in adolescents, and future studies should use designs that improve causal inferences.

We did not adjust for variables that might be on the causal pathway between breast development and depressive symptoms such as self-esteem, peer relations, family functioning, body mass index and substance abuse. It is possible that, because breast development is visible, these social and psychological factors alone explain the differential pattern of associations we observed. However, it is unlikely that any association between pubertal development and depression is explained by either social or biological factors alone. A complex interplay is much more likely. Our findings suggest that gonadal hormones could be more important to this interplay than adrenal hormones.

Puberty is associated with many social and psychological stressors experienced by males and females, that might increase risk of depression [37]. However, it is still unclear why more adolescent females experience depression than males. Our results are consistent with the hypothesis that changes in gonadal hormones (indicated by more advanced breast development) but not DHEA, could contribute to the increased incidence and prevalence of depression in female adolescents. This is supported by a previous study which found that increased estrogen was associated with depressive symptoms in adolescent girls, over and above associations with self-reported pubertal stages [29]. It is also consistent with a recent large cohort study reporting an increased risk of depression in women using hormonal contraceptives, especially adolescents [14]. As far as we are aware, only one study has directly measured female sex hormones in adolescence [29]. Our results suggest this could be an informative direction for future research. Hormonal mechanisms are extremely complex so direct measurements on repeated occasions are necessary in order to better unravel their influence on depressive symptoms.

It is possible that gonadal hormones increase adolescent girls’ cognitive vulnerability to stressors experienced in adolescence. There is some evidence from animal and human studies that estrogen and progesterone affect the brain and behavior, although these mechanisms are poorly understood and their influence on depression unclear [11,38–41]. For example, through effects on the hippocampus and hypothalamus, estrogens contribute to regulation of the hypothalamic-pituitary-adrenal axis which governs responses to stress [11,38]. Increased estrogen could also affect neural structures underlying mood, such as the amygdala and hippocampus, via dopamine and serotonin pathways (and through other mechanisms) [28,42].

Our longitudinal findings are that female adolescents at more advanced stages of breast development at age 14 continued to report more depressive symptoms later in adolescence. They were also at increased risk of meeting the criteria for clinical depression. These findings suggest that duration of exposure to sex hormones could be important [9]. Depression that fulfills criteria for DSM diagnosis may also take time to develop. If female sex hormones increases responsivity to stress, it may take time for stress to accumulate and depressive responses to develop.

The association between pubertal development and depressive symptoms is likely to involve a complex interplay of social, psychological and biological processes. Breast development also has important social and psychological consequences that are likely to contribute to the associations we observe with increased depressive symptoms. There is evidence that adolescents who develop earlier than their peers are more likely to be bullied and experience social isolation and more likely to engage in criminality, substance use, and early sexual behavior [43–45]. Future research should investigate how these social, psychological and biological influences combine to increase risk of female depression during and after puberty.

Our findings are consistent with the hypothesis that increased gonadal hormones are an endocrinological risk factor contributing to elevated depressive symptoms in females, but not males, throughout the life-course. Advanced breast development could therefore be a proxy marker for an endocrinological risk factor that is unique to females and contributes to the increased prevalence of depression in females relative to males. Social and psychological influences are also likely to be important in the association between advanced breast development and depressive symptoms. Further research on how female sex hormones might influence responses to stress, and interact with social and psychological factors, could facilitate our understanding of the sex difference in depression. Finally, our findings suggest that future studies should consider breast and pubic hair status as separate indicators of pubertal development because they are differentially associated with adolescent depression.

## Supporting information

S1 Table Associations between pubertal status (continuous exposure, coded 1 to 5) and DHEA (continuous outcome) in girls (N = 658) and boys (N = 511). (DOCX)

S2 Table Associations between DHEA at age 14.5 (exposure) and depressive symptoms at ages 14.5 and 17.5 in females and males. (DOCX)

S3 Table Cross-sectional associations between pubertal status (continuous exposure coded 1 to 5) and depressive symptoms (continuous outcome) in the complete case sample at age 14.5 (girls, n = 367; boys, n = 288). (DOCX)

S4 Table Longitudinal associations between pubertal status (continuous exposure variable coded 1 to 5) and depressive symptoms / diagnoses (outcome variable) in complete case sample (girls, n = 367; boys, n = 288). Longitudinal associations with depression diagnoses are presented for girls only (n = 338, those with lifetime depression diagnoses by the age of 14.5 excluded, n = 29).
(DOCX)
