# Supplementary material for: The association between pubertal status and depressive symptoms and diagnoses in adolescent females: A population-based cohort study
Source: PLoS One. 2018 Jun 18;13(6):e0198804. doi: 10.1371/journal.pone.0198804 (PMC6005470; doi:10.1371/journal.pone.0198804)
Supplement: S1 Table — (DOCX) [file pone.0198804.s001.docx]

S1 Table. Associations between pubertal status (continuous exposure, coded 1 to 5) and DHEA (continuous outcome) in girls (N=658) and boys (N=511).

| Pubertal status | Change^a^ in DHEA for a 1-stage increase in pubertal status (95% CI) p value | |
| --- | --- | --- |
|  | Before adjustment | Adjusted for other puberty measure^b^ |
| **Girls** |  |  |
| Breast | 0·11 (0·07 to 0·13) ·012 | 0·07 (-0·00 to 0·09) ·110 |
| Pubic hair | 0·14 (0·11 to 0·17) <·001 | 0·12 (0·09 to 0·15) ·005 |
| **Boys** |  |  |
| Genital | 0·14 (0·13 to 0·15) ·002 | 0·04 (-0·02 to 0·06) ·441 |
| Pubic hair | 0·19 (0·17 to 0·20) <·001 | 0·19 (0·17 to 0·20) ·001 |

^a^Change is represented by unstandardized regression coefficients.

^b^In models with breast status as the initial exposure variable, adjustment was made for pubic hair status. In models with pubic hair status as the initial exposure variable, adjustment was made for breast status in girls and genital status in boys.
