# Supplementary material for: The association between pubertal status and depressive symptoms and diagnoses in adolescent females: A population-based cohort study
Source: PLoS One. 2018 Jun 18;13(6):e0198804. doi: 10.1371/journal.pone.0198804 (PMC6005470; doi:10.1371/journal.pone.0198804)
Supplement: S2 Table — (DOCX) [file pone.0198804.s002.docx]

S2 Table. Associations between DHEA at age 14.5 (exposure) and depressive symptoms at ages 14·5 and 17·5 in females and males.

| DHEA | Females | | | | | |
| --- | --- | --- | --- | --- | --- | --- |
|  | Age 14·5 (N=487) | | | Age 17·5 (N=445) | | |
|  | coefficient | 95% CI | p | coefficient | 95% CI | P |
| Unadjusted | 3.15 | -.35 to 6.66 | 0·078 | 2.07 | -1·36 to 5.50 | 0.237 |
| Adjusted* | 2·12 | -1.32 to 5·56 | 0·227 | 0.35 | -2.70 to 3.41 | 0·820 |
|  | Males | | | | | |
| DHEA | Age 14·5 (N=355) | | | Age 17·5 (N=308) | | |
|  | coefficient | 95% CI | p | coefficient | 95% CI | P |
| Unadjusted | 0.90 | -2.34 to 4.14 | 0·584 | 0.63 | -2·66 to 3.92 | 0·707 |
| Adjusted* | 0.47 | -2.75 to 3.69 | 0·774 | -0.46 | -3.31 to 2.38 | 0·749 |

*Models at age 14.5 were adjusted for age and body mass index (BMI). Models at age 17.5 were adjusted for age, BMI and depressive symptoms at age 14.5.
