# Supplementary material for: The association between pubertal status and depressive symptoms and diagnoses in adolescent females: A population-based cohort study
Source: PLoS One. 2018 Jun 18;13(6):e0198804. doi: 10.1371/journal.pone.0198804 (PMC6005470; doi:10.1371/journal.pone.0198804)
Supplement: S4 Table — Longitudinal associations with depression diagnoses are presented for girls only (n = 338, those with lifetime depression diagnoses by the age of 14.5 excluded, n = 29). (DOCX) [file pone.0198804.s004.docx]

S4 Table. Longitudinal associations between pubertal status (continuous exposure variable coded 1 to 5) and depressive symptoms / diagnoses (outcome variable) in complete case sample (girls, n=367; boys, n=288). Longitudinal associations with depression diagnoses are presented for girls only (n=338, those with lifetime depression diagnoses by the age of 14·5 excluded, n=29).

| Linear regressions for depressive symptoms | Change^a^ in depressive symptoms for a 1-stage increase in pubertal status (95% CI) p value | | |
| --- | --- | --- | --- |
|  | Girls | | Boys |
|  | Breast status | Pubic hair status | Pubic hair status |
| Model 1: Unadjusted | 1.56 (.38 to 2.75) .010 | .20 (-1.15 to 1.54) .772 | .93 (-.21 to 2.07) .111 |
| Model 2: Model 1 adjusted for other puberty measure^b^ | 1.59 (.38 to 2.79) .010 | -.14 (-1.50 to 1.22) .843 | .53 (-.85 to 1.91) .447 |
| Model 3: Model 2 adjusted for age and pubertal timing^c^ | 1.82 (.59 to 3.06) .004 | -.11 (-1.48 to 1.25) .869 | .54 (-.85 to 1.93) .442 |
| Model 4: Model 3 adjusted for possible confounders^d^ | 1.62 (.36 to 2.88) .012 | -.40 (-1.78 to .98) .571 | .27 (-1.11 to 1.65) .699 |
| Logistic regressions for depressive diagnoses | Odds ratio (95% CI) p value | | |
|  | Breast status | Pubic hair status | Pubic hair status |
| Model 1: Unadjusted | 1.39 (.97 to 1.99) .076 | 1.04 (.70 to 1.55) .844 | n/a |
| Model 2: Model 1 adjusted for other puberty measure^b^ | 1.39 (.97 to 2.01) .076 | .98 (.65 to 1.47) .908 | n/a |
| Model 3: Model 2 adjusted for age and pubertal timing^c^ | 1.38 (.95 to 2.00) .095 | .97 (.65 to 1.47) .902 | n/a |
| Model 4: Model 3 adjusted for possible confounders^d^ | 1.37 (.93 to 2.02) .107 | .96 (.63 to 1.45) .834 | n/a |

^a^Change is represented by the unstandardized regression coefficient.

^b^In models with breast status as the initial exposure variable, adjustment was made for pubic hair status. In models with pubic hair status as the initial exposure variable, adjustment was made for breast status in girls and genital status in boys.

^c^Pubertal timing was included in models for girls only because this measure was unavailable for boys.

^d^Other possible confounders were childhood adversity, social class, maternal education, and BMI.
